# A Case for Doxycycline as an Effective Treatment for Confluent and Reticulated Papillomatosis (CARP)

**DOI:** 10.1155/2023/6397272

**Published:** 2023-05-13

**Authors:** Aishwarya Sharma, Deborah Moon, Dong Joo Kim

**Affiliations:** ^1^School of Medicine and Health Sciences, University of North Dakota, Grand Forks, ND 58203, USA; ^2^Department of Dermatology, University of California, Irvine, CA 92697, USA; ^3^Department of Internal Medicine, University of North Dakota, Fargo, ND 58102, USA; ^4^Department of Dermatology, Essentia Health, Fargo, ND 58103, USA

## Abstract

Confluent and reticulated papillomatosis (CARP) is a rare keratinization disorder that presents with asymptomatic, reticulated papules coalescing into plaques, which adversely affect, most often, young black persons. Minocycline is considered the drug of choice, but it is not without its host of potential side effects, including drug hypersensitivity, drug-induced lupus/vasculitis/hepatitis, blue-gray skin hyperpigmentation, acute eosinophilic pneumonia, pseudotumor cerebri, and vestibular instability, among others. Alternatively, doxycycline might be considered as another first-line agent for CARP as it can effectively clear lesions while offering a more favorable side effect profile in select patients. Herein, we present a case of CARP successfully resolved with doxycycline after a protracted treatment history of topical and oral antifungal medications for suspected tinea versicolor (TV).

## 1. Introduction

Confluent and reticulated papillomatosis (CARP) of Gougerot and Carteaud is a chronic disorder of keratinization that typically presents around puberty and disproportionately affects young, black adults [1]. Characteristic lesions present as scaly, brown macules and papules that coalesce into reticulated patches and plaques on the neck, back, and flexural surfaces [2]. Minocycline has historically been considered the treatment of choice for CARP; however, given its side effect profile, including but not limited to hyperpigmentation, drug-induced hypersensitivity syndrome, drug-induced lupus, dyspigmentation, and vestibular dysfunction, alternative therapies with a more favorable side effect profile, such as doxycycline, may be worth considering [3]. While there have been reports of successful responses to doxycycline, it is still not considered a first-line agent for CARP. We present a case of CARP cleared with doxycycline in an African American male who had previously been treated unsuccessfully for tinea versicolor (TV).

## 2. Case Report

**Figure 1:**
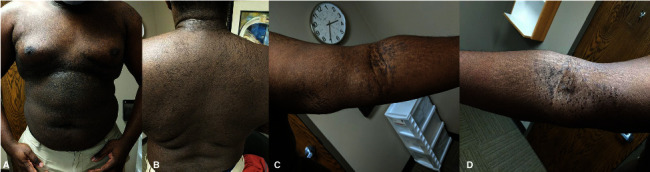
Confluent and reticulated papillomatosis, initial clinical presentation. Brown confluent papules and plaques on the (a) chest, (b) back, (c) left arm, and (d) right arm.

**Figure 2:**
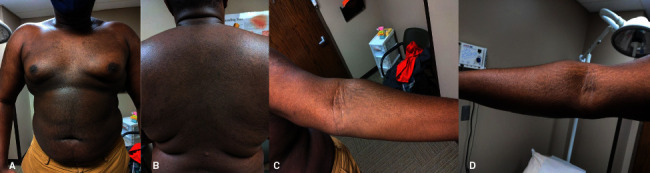
Confluent and reticulated papillomatosis, 6-week follow-up. Clearance achieved after 6 weeks of oral doxycycline therapy on the (a) chest, (b) back, (c) left arm, and (d) right arm.

A 26-year-old otherwise healthy African American male presented with well-circumscribed red-brown papules coalescing into reticulated plaques with minimal to no scale scattered on his neck, chest, back, antecubital fossa, and extremities (Figure 1) ongoing for the past 6 years. He had been receiving treatment for TV with topical and oral antifungals, including an extensive trial of oral itraconazole, without any success. A clinical diagnosis of CARP was favored, and the patient was started on doxycycline 100 mg by mouth twice daily, with significant improvement noted at six-week follow-up (Figure 2). The patient was continued on doxycycline 100 mg twice daily for 6 more weeks (12 weeks continuous treatment) and then gradually tapered off over 3 months (100 mg daily for 1 month, then 100 mg every other day for 1 month, then 100 mg weekends for 1 month, then off), achieving sustained clearance of his rash after 6 months.

## 3. Discussion

CARP is a rare disorder of keratinization that presents with asymptomatic, reticulated, sometimes warty papules coalescing into plaques affecting the trunk, axilla, neck, and flexor surfaces more commonly in young women and blacks. CARP is a clinical diagnosis; however, a skin biopsy showing histopathological features of hyperkeratosis, acanthosis, papillomatosis, and superficial perivascular lymphocytic infiltrates can help to confirm the diagnosis [2]. Characteristic histological features include club-shaped, bulbous epidermal rete ridges that extend into the papillary dermis with pigment at their tips. Oral antibiotics remain the cornerstone of therapy.

Minocycline is a tetracycline-class antibiotic that is often considered first-line treatment since it has been found to completely clear skin eruptions; however, recurrence in the subsequent months following cessation of therapy is common [1]. Minocycline-associated toxicities, compared to other tetracycline-class antibiotics, include drug hypersensitivity, drug-induced lupus/vasculitis/hepatitis, blue-gray skin hyperpigmentation, acute eosinophilic pneumonia, pseudotumor cerebri, and vestibular instability [3]. While the absolute risk is comparatively low, there is an 8.5-fold increased risk of minocycline-induced lupus [4]. Conversely, doxycycline—an equally long-acting tetracycline antibiotic—has arguably a more favorable side effect profile including GI upset and photosensitivity.

Doxycycline 100 mg twice daily for 3 months has been an effective alternative in treating CARP, but its use remains limited as the second-line treatment in isolated cases [3]. Response rates in the literature have been variable, with one retrospective study in southern India suggesting a less robust response to doxycycline compared to minocycline [5]. This is in contrast to a retrospective review showing 4 of 4 patients treated with doxycycline had complete clearance after 4–6 weeks of treatment [6]. This is also noteworthy, as a 30-year retrospective review identified 39 patients with CARP of whom 20 percent did not achieve clearance using minocycline [1].

Furthermore, studies exploring differences in demographics and response rates may provide additional insight. The odds of CARP are increased 4-16-fold in patients who are black, overweight/obese, or diagnosed with acanthosis nigricans [6]. Given its clinical mimic with TV, identification and treatment of CARP are more likely to be underdiagnosed in skin of color (SOC) [7]. The paucity of literature on skin diseases affecting the Afro-Caribbean population—CARP being amongs the top 12—increases the necessity for further clinical suspicion when analyzing hypo- and hyperpigmented lesions in SOC patients [8]. Proposed diagnostic criteria for CARP includes (a) scaly brown macules and patches with some reticulated and papillomatous, (b) upper trunk and neck involvement, (c) negative fungal staining of scales, (d) no clearance using antifungal treatment, and (e) response to tetracycline [1]. White patients have been the primary focus of previous studies regarding CARP with only isolated cases being reported in SOC patients treated with minocycline [9]. This further highlights the unique nature of our case report and the necessity for consideration of doxycycline as first-line therapy.

Although less frequently used, clinicians should be aware of the effectiveness and limited adverse effects of doxycycline compared to minocycline for complete resolution of CARP. Doxycycline has a lower incidence of systemic adverse effects, especially in patients with SOC. Given the risk of recurrence, long-term follow-up may be needed after treatment of CARP with doxycycline. Increased reporting in the literature of response rates to doxycycline may have practical implications on clinical management of CARP and may bring doxycycline to the forefront as a first-line therapy option for the appropriately selected patient considering its potentially more favorable side effect profile.

## Data Availability

No underlying data were collected or produced in this study.

## Abbreviations

CARP: Confluent and reticulated papillomatosis
AN: Acanthosis nigricans
SOC: Skin of color
TV: Tinea versicolor.
